# Assessment of Genetic Associations between Common Single Nucleotide Polymorphisms in RIG-I-Like Receptor and IL-4 Signaling Genes and Severe Respiratory Syncytial Virus Infection in Children: A Candidate Gene Case-Control Study

**DOI:** 10.1371/journal.pone.0100269

**Published:** 2014-06-20

**Authors:** Nico Marr, Aaron F. Hirschfeld, Angie Lam, Shirley Wang, Pascal M. Lavoie, Stuart E. Turvey

**Affiliations:** Department of Pediatrics, University of British Columbia, Child and Family Research Institute, Vancouver, British Columbia, Canada; University of Iowa, United States of America

## Abstract

The majority of cases of severe pediatric respiratory syncytial virus (RSV) infection occur in otherwise healthy infants who have no identifiable risk factors, suggesting that additional subclinical factors, such as population genetic variation, influence the course of RSV infection. The objective of this study was to test if common single nucleotide polymorphisms (SNPs) in genes encoding for immune signalling components of the RIG-I-like receptor (RLR) and IL-4-signalling pathways affect the outcome of RSV infection in early life. We genotyped 8 SNPs using allele-specific probes combined with real-time PCR. Each of the SNPs tested had previously been established to have a functional impact on immune responsiveness and two of the SNPs in the *IL4* and *IL4R* genes had previously been associated with severe RSV bronchiolitis. Association with susceptibility to severe RSV infection was tested by statistically comparing genotype and allele frequencies in infants and young children hospitalized with severe RSV bronchiolitis (n = 140) with two control groups—children who tested positive for RSV but did not require hospitalization (n = 100), and a general population control group (n = 285). Our study was designed with sufficient power (>80%) to detect clinically-relevant associations with effect sizes ≥1.5. However, we detected no statistically significant differences in allele and genotype frequencies of the investigated SNPs between the inpatient and control groups. To conclude, we could not replicate the previously reported association with SNPs in the *IL4* and *IL4R* genes in our independent cohort, nor did we find that common SNPs in genes encoding for RLRs and the downstream adapter MAVS were associated with susceptibility to severe RSV infections. Despite the existing evidence demonstrating a functional immunological impact of these SNPs, our data suggest that the biological effect of each individual SNP is unlikely to affect clinical outcomes of RSV infection.

## Introduction

Human respiratory syncytial virus (RSV) is the most important respiratory pathogen of early life. In developed countries, RSV infection accounts for more than half of all cases of acute respiratory tract infections and influenza-like illnesses in infants and young children [1], [2]. Recent estimates suggest that each year, up to 234,000 children under age 5 die from clinical complications due to RSV infections worldwide [3], [4]. In addition, severe respiratory virus infection in early life is an important risk factor for the development of asthma [5].

A complex combination of environmental, pathogen and host genetic factors determines both susceptibility to pathogens and the course of infection. Nevertheless, infectious diseases have an inherited element and individuals with different genetic backgrounds respond differently to particular infections. Adoption studies, which effectively separate genetic and environmental confounders, have indicated a substantial genetic effect involved in susceptibility to infection. For example, the early death of a biological parent from infection increased the risk of death of the child from an infectious disease nearly six-fold. In contrast, the death of an adoptive parent from an infectious disease had no significant effect on the adoptees' risk of such a death [6], [7].

A variety of clinical risk factors are associated with severe RSV infection—premature birth, bronchopulmonary dysplasia, congenital heart disease, and immunodeficiency [1]. Nevertheless, the majority of severe RSV infections occur in infants born at term who were previously healthy [8], [9], [10]. This strongly suggests that additional influences, such as genetic variability of the host, contribute to disease severity. The importance of the contribution of host genetics in RSV immunopathogenesis was further highlighted by twin studies, revealing that identical twin pairs had significantly higher concordance rates in the susceptibility to severe RSV infection when compared with fraternal twin pairs [11]. These findings are consistent with genetic association and case-control studies suggesting a role of familial susceptibility to severe viral respiratory infections in early life [12], [13], [14], [15].

The goal of this study was to test if common single nucleotide polymorphisms (SNPs) in genes coding for immune signalling components of the RIG-I-like receptor (RLR) and IL-4-signalling pathways affect the outcome of RSV infection. We chose to employ this targeted candidate gene approach because there is strong evidence that both pathways play a critical role in RSV immunopathogenesis. The importance of the ubiquitously expressed cytosolic receptor, RIG-I, in the replication-dependent recognition of RSV in airway epithelial cells (AEC) has been well established [16] and we have recently demonstrated a role of RIG-I in the innate antiviral response to RSV in human plasmacytoid dendritic cells [8]. In addition, its structural homolog MDA5 appears to play a non-redundant role, at the least in AEC [16], [17]. The innate antiviral response elicited through these cytosolic PRRs is critical in restricting RSV replication in the respiratory tract [18], [19]. Similarly, IL-4-mediated signaling appears to play a pathological role later during RSV infection [20]. RSV infection in early life has been shown to be associated with Th2-biased immune responses following RSV infection [21], [22]. This is likely due to increased IL-4Rα expression levels in neonatal CD4+T cells, which triggers increased immunopathology and perhaps also long-term changes in normal immune function (e.g. asthma development) following neonatal RSV infection [20], [23].

We investigated the role of common, non-synonymous coding SNPs and one promoter SNP in the genes encoding RIG-I and MDA5, its specific adaptor protein MAVS, as well as the genes encoding IL-4 and the IL-4 receptor alpha (IL-4Rα) (Table 1). All SNPs that we analyzed in this present study have been reported to play a functional role in immune signalling in cell models *in vitro*, in antibody responses to vaccination, and/or in the manifestation of a variety of human illnesses *in vivo*, ranging from infections with other RNA viruses to non-communicable diseases such as type 1 diabetes or systemic lupus (for details see Table 1). The *IL4* promoter and *IL4R* gene SNPs examined in this study have been reported to be associated with severe RSV bronchiolitis in children in Korea and the Netherlands, respectively [24], [25]. However, another independent study of a cohort of children in Germany [26] did not find an association between this *IL4* promoter SNP and outcomes of pediatric RSV infection thus warranting further confirmation using our cohort of children in Canada.

**Table 1:** SNPs analyzed in this study.

Gene name (protein)	SNP ID	Position	Nucleotide Change	Amino Acid Change	Evidence for functional role
*DDX58* (RIG-I)	rs10813831	CDS	C→T	Arg7Cys	Increased *DDX58* and *IFNB1* transcription in Newcastle disease virus-infected primary DCs [44]; allele dose-related decrease of antibody levels after rubella vaccination [46]
	rs17217280	CDS	T→A	Asp580Glu	Reduced IFN-β-, IRF-3-, NF-κB-dependent reporter gene activation in HEK 293T or BEAS-2B cells at baseline and upon Influenza virus A challenge [45]
					
*IFIH1* (MDA-5)	rs3747517	CDS	A→G	His843Arg	Type 1 diabetes [51]
	rs1990760	CDS	G→A	Ala946Thr	Type 1 diabetes [50]
					
*MAVS* (MAVS)	rs17857295	CDS	C→G	Gln93Glu	Subgroup of systematic lupus erythematoses patients with renal nephritis [47]
	rs7269320	CDS	C→T	Ser409Phe*	Subgroup of systematic lupus erythematosus patients with arthritis [47]
					
*IL4* (IL-4)	rs2243250	promoter	C→T	N/A	Hospitalization due to RSV bronchiolitis [24], [25]
					
*IL4R* (IL4-Rα)	rs1801275	CDS	A→G	Gln576Arg*	Hospitalization due to RSV bronchiolitis in children >6 months old [24]

CDS, coding sequence; *transcript variant 1.

## Materials and Methods

### DNA sample collection and genotyping

**Figure 1:**
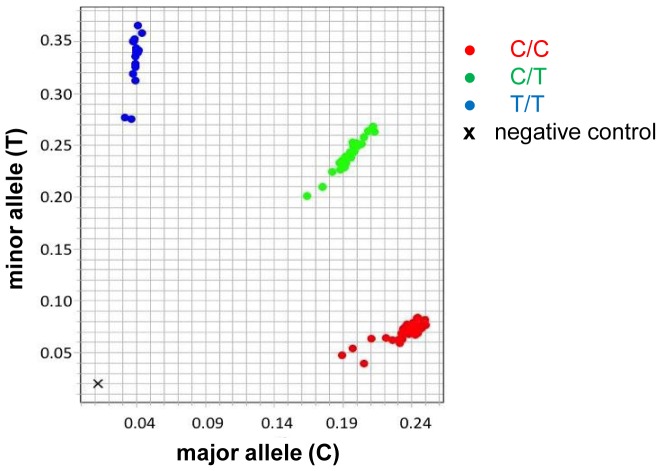
Representative data set (*IL4* C→T, rs2243250) analyzed by real-time PCR assay using allele-specific probes.

We obtained de-identified genomic DNA samples from nasopharyngeal washes (NPW) of children (n = 240) visiting the Emergency Room at Children's & Women's Health Centre of British Columbia (Vancouver, Canada) and who tested positive for RSV infection by direct immunofluorescence assays as previously described [27]. We differentiated between children who required hospitalization due to severe RSV bronchiolitis (inpatients, n = 140) and children with confirmed RSV infection who did not require hospital admission (outpatients, n = 100). Moreover, we collected genomic DNA samples of a general population control group (n = 285) from umbilical cord blood of healthy term neonates born by scheduled Cesarean sections at the same institution. DNA collection was approved by the University of British Columbia's Clinical Research Ethics Board. Specifically, written parental consent was obtained for umbilical cord blood collections of healthy term neonates providing genomic DNA samples of the general population control group. For subjects who tested positive for RSV, DNA was isolated from de-identified NPW samples where an individual's identifying information had been removed, and in this specific circumstance consent was not obtained. Our institutional ethics committee has approved DNA collection from all subjects, including DNA collection from de-identified NPW samples where consent was not obtained. Genotyping was performed by real time PCR assays using allele-specific Taqman probes (Fig. 1) as previously described [27], [28].

### Statistical analysis

Genotype and allele frequencies in the outpatient group and the general population control group were compared separately to the inpatient group using Pearson Chi-Square tests, or when one of the genotype or allele counts in any given group was less than 5 we applied the Fisher Exact Probability tests. Allelic association was tested by comparing the allele counts in the case and control groups using 2×2 contingency tables. Genotypic association was tested by comparing the genotype counts using 2×3 contingency tables (general model with 2 degrees of freedom), as well as by comparing the genotype counts after combining the homozygous and heterozygous genotype counts carrying the minor allele and using 2×2 contingency tables (dominant model) [29]. *P* values≤0.05 and odds ratios (OR) with a 95% confidence interval (CI) ≠1 were considered statistically significant. Statistics were computed using utilities available at the VassarStats website for statistical computation (http://vassarstats.net). Power calculations were made using CaTS software [30].

## Results

In the present study we used a targeted candidate gene approach and analyzed SNPs in genes encoding RIG-I and MDA-5, its downstream adapter MAVS, as well as IL-4 and IL-4Rα, since there is evidence that these immune signaling components play a pivotal role during RSV immunopathogenesis [8], [16], [20]. We chose to focus on common SNPs where a functional role had been reported in independent studies (Table 1). SNPs were considered common if they had a global minor allele frequency (MAF) of >5% (i.e. MAF reported in dbSNP from the current 1000Genome default population including 1094 worldwide individuals [31]). All synonymous SNPs as well as rare SNPs (i.e. SNPs with low or undetermined MAF, or rare mutations) were excluded from our analysis since they are less likely to play a biological role and/or are unlikely to affect outcomes at the population level, respectively. In the case of multiple variants in strong linkage disequilibrium (LD), only one SNP was analyzed.

**Figure 2:**
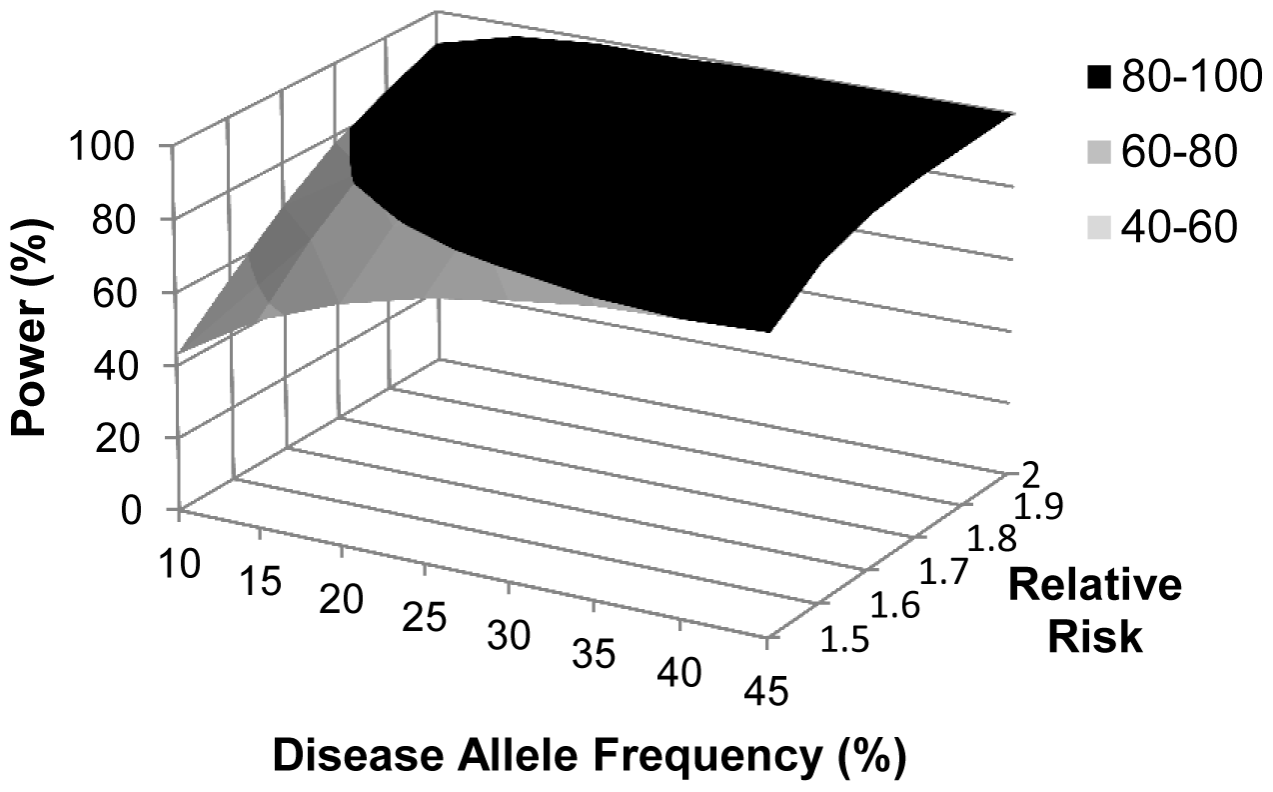
Statistical power to detect associations in our case-control study (n = 140 cases and 285 controls). Power calculations were done for combinations of disease allele frequencies between 10% and 45% (in increments of 5%) and relative risks between 1.5 and 2 (in increments of 0.1) using a multiplicative model of penetrance and a significance level of 0.05. Disease prevalence was set at 2%. Area shaded in black indicates ≥80 power.

Using these selection criteria, we analyzed 8 SNPs in total (Table 1). To test whether any of these SNPs may affect the outcome of RSV infection in children, we genotyped 140 children who were hospitalized with confirmed RSV infection (inpatients), 100 children who tested positive for RSV infection but had less severe disease manifestations and therefore did not require hospital admission (outpatients), as well as a general population control group (n = 285) of healthy term neonates. Genotyping was performed by real-time PCR assays using allele-specific TaqMan probes (Fig. 1). Based upon the sample size of our inpatient and population control group, we estimated that our study had ≥80% power to detect clinically-actionable associations; specifically, with relative risks of ≥1.9, ≥1.7, ≥1.6 and ≥1.5 for disease allele frequencies of 10–14%, 15–20%, 21–36%, and ≥37%, respectively (Fig. 2). All analyzed SNPs had a genotyping call rate between 96 and 100% (Table 2).

**Table 2:** Case-control association analysis between SNPs in RIG-like receptor and IL-4 signaling genes and severe RSV infection.

Gene and SNP ID	call rate % (n)	Allele Freq. % (n)		*P_a_*	OR (95% CI)	Genotype Freq. % (n)			*P_b_*	*P_c_*	OR (95% CI)
***DDX58***											
** rs10813831**		C	T			CC	CT	TT			
Population Control	99 (281)	79 (444)	21 (118)	0.47	0.87 (0.61–1.26)	64 (179)	31 (86)	6 (16)	0.25	0.86	0.97 (0.63–1.48)
Outpatients	100 (100)	84 (167)	17 (33)	0.51	1.17 (0.73–1.90)	71 (71)	25 (25)	4 (4)	0.33*	0.29	1.35 (0.77–2.35)
Inpatients	99 (138)	81 (224)	19 (52)			64 (89)	33 (46)	2 (3)			
** rs17217280**		A	T			AA	AT	TT			
Population Control	99 (283)	89 (502)	11 (64)	0.68	0.91 (0.57–1.44)	78 (221)	21 (60)	1 (2)	0.57	0.53	0.85 (0.51–1.41)
Outpatients	100 (100)	90 (180)	10 (20)	0.89	1.04 (0.57–1.90)	80 (80)	20 (20)	0 (0)	0.58*	0.89	0.96 (0.50–1.82)
Inpatients	100 (140)	90 (251)	10 (29)			81 (113)	18 (25)	1 (2)			
***IFIH1***											
**rs3747517**		G	A			GG	GA	AA			
Population Control	98 (279)	57 (317)	43 (241)	0.40	0.88 (0.66–1.18)	34 (95)	46 (127)	20 (57)	0.42	0.22	0.77 (0.50–1.17)
Outpatients	98 (98)	59 (116)	41 (80)	0.89	0.97 (0.67–1.41)	43 (42)	33 (32)	24 (24)	0.54	0.68	1.12 (0.66–1.89)
Inpatients	98 (137)	60 (164)	40 (110)			40 (55)	39 (54)	20 (28)			
** rs1990760**		A	G			AA	AG	GG			
Population Control	98 (278)	45 (252)	55 (304)	0.21	0.83 (0.62–1.11)	26 (72)	39 (108)	35 (98)	0.43	0.52	0.86 (0.54–1.36)
Outpatients	98 (98)	52 (101)	48 (95)	0.74	1.06 (0.74–1.54)	34 (33)	36 (35)	31 (30)	0.58	0.43	1.25 (0.71–2.19)
Inpatients	96 (135)	50 (135)	50 (135)			29 (39)	42 (57)	29 (39)			
***MAVS***											
** rs17857295**		C	G			CC	CG	GG			
Population Control	99 (283)	67 (382)	33 (184)	0.81	1.04 (0.76–1.41)	49 (139)	37 (104)	14 (40)	0.96	0.92	1.02 (0.68–1.54)
Outpatients	100 (100)	66 (132)	34 (68)	0.89	0.97 (0.66–1.43)	45 (45)	42 (42)	13 (13)	0.65	0.59	0.87 (0.52–1.45)
Inpatients	99 (138)	67 (184)	33 (92)			49 (67)	36 (50)	15 (21)			
** rs7269320**		C	T			CC	CT	TT			
Population Control	99 (283)	85 (480)	15 (86)	0.79	0.95 (0.63–1.42)	72 (205)	25 (70)	3 (8)	0.74	0.63	0.89 (0.56–1.42)
Outpatients	100 (100)	86 (172)	14 (28)	0.89	1.04 (0.62–1.75)	76 (76)	20 (20)	4 (4)	0.97*	0.81	1.08 (0.59–1.96)
Inpatients	99 (138)	86 (236)	14 (40)			75 (103)	22 (30)	4 (5)			
***IL4***											
** rs2243250**		C	T			CC	CT	TT			
Population Control	100 (284)	65 (368)	35 (200)	0.36	0.87 (0.64–1.18)	46 (130)	38 (108)	16 (46)	0.69	0.42	0.84 (0.54–1.27)
Outpatients	100 (100)	64 (128)	36 (72)	0.36	0.84 (0.57–1.23)	48 (48)	32 (32)	20 (20)	0.45	0.76	0.92 (0.55–1.55)
Inpatients	96 (136)	68 (185)	32 (87)			50 (68)	36 (49)	14 (19)			
***IL4R***											
** rs1801275**		A	G			AA	AG	GG			
Population Control	99 (283)	77 (434)	23 (132)	**0.05**	0.69 (0.48–1.00)	59 (168)	35 (98)	6 (17)	0.12	0.11	0.71 (0.46–1.08)
Outpatients	100 (100)	76 (151)	25 (49)	0.06	0.65 (0.41–1.02)	57 (57)	37 (37)	6 (6)	0.14*	0.10	0.64 (0.38–1.09)
Inpatients	99 (138)	83 (228)	17 (48)			67 (93)	30 (42)	2 (3)			

*P_a_*: uncorrected *P* values vs. inpatient group for allelic association;

*P_b_*: uncorrected *P* values vs. inpatient group for genotypic association (2 degrees of freedom);

*P_c_*: uncorrected *P* values vs. inpatient group for genotypic association after combining the homozygous and heterozygous genotype counts carrying the minor allele (dominant model);

*Indicates *P* values computed by Fisher exact probability tests; OR: Odds Ratios; 95%CI: 95% confidence intervals.

To test for associations in our candidate gene case-control study, we performed χ^2^ tests or Fisher exact probability tests when appropriate, applying three standard models of disease penetrance. First we tested for allelic association by comparing the allele counts in the control groups with that in the inpatient group (multiplicative model). In addition, we assessed genotypic association, assuming that each of the genotypes has an independent association with disease (2 degrees of freedom). To account for an alternative model of disease penetrance, we also applied a dominant model by combining the homozygous and heterozygous genotype counts carrying the minor allele [29]. We separately compared the allele and genotype frequencies of each SNP in the inpatient group with that in our two control groups, namely (i) outpatients with confirmed RSV infection, or (ii) a population control group of term infants who were recruited at the same institution, and applied the three models of disease penetrance as outlined above for each comparison. As shown in Table 2, we found no statistically significant associations between a particular genotype or allele frequency and hospitalization due to RSV bronchiolitis, regardless of the genetic model, and whether the inpatient group was compared to the outpatient group, or the general population control group. Our analysis only revealed a modest overrepresentation of the minor allele of the IL-4Rα SNP among the general population control group (uncorrected *P* value = 0.05; OR 0.69; 95%Cl 0.48–1.00), which was not statistically significant after correction for multiple comparisons. Of note, Hoebee *et al*. [24] had previously reported an association between the *IL4* SNP tested in our study and severe RSV infection, which appeared to be more robust when children <6 month of age, or those with recurrent wheezing, cardiac or lung disease were excluded. However, comparison of our inpatient group with our outpatient group using the three standard models of disease penetrance as outlined above did not reveal a statistically significant association between any of the SNPs tested here and severe RSV infection after stratification of the RSV-infected inpatients and outpatients in a group ≤6 month of age and a group >6 months of age (Tables S1A and S1B). DNA samples of our population control group were obtained from cord blood (i.e. at birth) and were therefore not used to separately assess the effect of age as a co-variable. In our study, we did not collect additional clinical data, thus precluding inclusion of other risk factors as co-variables in our analysis. De-identified genotype data and related metadata (i.e. ages, study group) are listed in Table S2. Further inquiries for additional data should be made to either SET or NM.

## Discussion

Today the only effective option to reduce the risk for severe RSV disease in infants is passive immunization with palivizumab (commonly referred to as RSV immunoprophylaxis), which is given by monthly intramuscular injections during the RSV season. However, the high costs of this therapy and the lack of useful indicators restricts its use to relatively few high-risk children, such as infants with congenital abnormalities or those born very early in gestation [32], [33]. There is a pressing need to establish novel indicators for the identification of children at high risk of developing severe RSV infections who do not meet the current criteria for RSV immunoprophylaxis. Genetic variants, such as common SNPs in genes coding for pattern recognition receptors or other crucial immune signalling and defence components, are an attractive target [34]. Gene SNPs have been shown to play a role in the clinical manifestations and outcome of a variety of infectious and immune-mediated human diseases; most notable examples include hepatitis C virus infections, Crohn's disease, and type 1 diabetes [35], [36], [37]. However, there is only limited information available about the role of SNPs and the risk of developing severe RSV infection in early life. Candidate gene association studies have largely focused on SNPs in the genes encoding Toll-like receptor (TLR) 4 or surfactant proteins A and D (SP-A, SP-D) and outcome of RSV infection [34]. However, independent studies assessing associations between *TLR4* SNPs and susceptibility to RSV infection have led to contradictory results [27], [38], [39], [40], and the role of this pattern recognition receptor (PRR) in RSV pathogenesis remains controversial [16]. Moreover, reported relative risks associated with SP-A and SP-D variants [34], [41] are not robust enough to justify their clinical use for the identification of at-risk children who may benefit from, but currently do not qualify for, RSV immunoprophylaxis.

Our results suggest that none of the common SNPs in the *IL4* and *IL4R* genes tested in this study appear to play a clinically-significant biological role in the outcome of pediatric RSV infection on its own. Although we found a modest overrepresentation of the minor allele for the *IL4R* SNP (rs1801275) among the general population control, this was not statistically significant after correcting for multiple testing. In addition, this modest overrepresentation of the *IL4R* SNP among the control group was in the opposite direction compared to a previous genetic association study by Hoebee *et al*. [24], who found this SNP to be associated with severe RSV infection in a subpopulation of children hospitalized at >6 month of age. The *IL4* promoter SNP tested in our study was associated with severe RSV infection in two association studies of children in Korea and the Netherlands, respectively [24], [25]. However, the lack of an association in our Canadian cohort is consistent with the finding of a previous study in a German cohort of children with severe RSV infection, who also showed no significant difference in the minor allele frequency in comparison to control subjects [26]. This is despite evidence of a functional role of this SNP in atopic disorders by contributing to enhanced IgE synthesis [42], [43]. The discrepancy between the results of genetic association studies focused on the *IL4* promoter SNP and severe RSV infection could have various reasons, including ethnic diversity, choice of control groups, differences in study design and statistical analysis. For example, Hoebee *et al*. [24] obtained DNA samples from children hospitalized with RSV and their parents, thereby combining a case-control study with a transmission/disequilibrium test. It should be noted that p-values reported by Hoebee *et al*. [24] and Choi *et al*. [25] were not corrected for multiple testing. If conservative correction for multiple testing of 3 or more SNPs in the same cohort is applied, associations reported in both studies become marginal.

Similarly, although there is evidence for a functional role of non-synonymous SNPs in genes encoding for RIG-I, MDA5, and its downstream adapter MAVS, we did not find an association of these SNPs with severe RSV infection. The two *DDX58* (*RIG-I*) SNPs analyzed in our study have been shown to cause modest changes in the innate antiviral response of human dendritic cells [44], HEK 293T or BEAS-2B cells [45]. One of the SNPs (rs10813831, Arg7Cys) in the gene encoding RIG-I was also found to be associated with an allele dose-related decrease in rubella antibody levels, further supporting a functional role of this variant [46]. The SNPs in the gene coding for MAVS appeared to be associated with systemic lupus erythematosus in the Chinese population, albeit only in patients with renal nephritis and arthritis, respectively [47]. Another study did not find an association between either of the SNPs in the genes coding for RIG-I and MAVS and outcome of hepatitis C virus infections [48]. Other variants with more deleterious effects on RIG-I [45] and MAVS [49] expression or function are rare, and therefore are unlikely to play a significant role in the outcome of RSV infection at the population level. Genome-wide association studies suggested a role of common variants in the gene encoding MDA5 in type I diabetes, which was later validated in independent cohorts [37], [50], [51]. Association studies linking SNPs in the gene encoding MDA5 with the outcome of viral infection are largely lacking. This is perhaps because MDA5 was initially believed to play a role in innate immune recognition of only a subset of RNA viruses [52]. However, more recent reports suggest that several viruses, including ssRNA viruses, are sensed by both RIG-I and MDA5, thereby engaging non-redundant signaling pathways [16], [53], [54].

It is important to point out strengths and limitations of our study. In our candidate gene case-control study, we applied three different models of disease penetrance and separately compared genotype and allele frequencies in our inpatient group with severe RSV disease (i.e. case group) with two different control groups, namely (i) a general population control group of term infants born at the same institution and (ii) a group of outpatients who visited the Children's & Women's Health Centre of British Columbia and were tested positive for acute RSV infection but did not require hospitalization (i.e. had less severe RSV disease). Comparison to each control group bears advantages and limitations over the other. Although our general population control group of term infants is characterized as a “low risk” group when considering all known clinical risk factors for severe RSV infection, this group likely includes some individuals who will go on to develop severe RSV infection requiring hospitalization. This is because even some previously healthy infants and young children develop severe RSV infection for reasons that remain unknown [8]. Nevertheless, the number of these individuals in our control group is expected to be very small and therefore, unlikely to skew the results of our genetic association study. Specifically, all individuals of our population control group were born by scheduled Cesarean section at term. Clinical conditions that are known to increase the risk factors for severe RSV disease among term infants, such as cardiac or lung disease, are relatively rare. Consequently, we estimate the rate of hospitalization due to RSV among our general population control group to be ≤2%, as reported for similar pediatric cohorts in other geographical regions [55]. The outpatient group which we used as a second control group lacks any children that required hospital admission. Nonetheless, one may consider this group as a medium rather than a low risk group, because all these children had symptomatic respiratory infection that prompted their caregivers and/or general physicians to bring them/to refer them to the Children's & Women's Health Centre of British Columbia.

In summary, our findings suggest that the SNPs analyzed in the present study are unlikely to significantly increase the risk of children developing severe RSV infection. These variants may still have minor effects on the outcome of RSV infection in infants and young children (RR≤1.4), a possibility that would require larger cohort size to prove. Nevertheless, the fact that various previously reported relative risks associated with common variants in host defense genes (e.g. *TLR4, IL4R*) and severe outcomes of pediatric RSV infection are generally low and that reported associations are often not reproducible in independent studies [34] highlights the need for more robust approaches for genetic susceptibility testing in the context of RSV infection and prophylaxis. Importantly, genetic variants exerting larger biological effects are generally too rare to justify clinical use. Future research is needed to test whether assessment of a combination of multiple common variants, rather than single risk alleles, may justify the use of genetic susceptibility testing to aid clinical decision-making for the purpose of selecting at-risk children for RSV immunoprophylaxis.

## Supporting Information

Table S1 **A and B: Case-control association analysis between SNPs in RIG-like receptor and IL-4 signaling genes and severe RSV infection in children ≤6 months of age (A) and in children >6 months of age (B).**
(XLSX)

Table S2 **De-identified genotype data and related metadata.**
(XLSX)
